# Adherence to inhalers and associated factors among adult asthma patients: an outpatient-based study in a tertiary hospital of Rajshahi, Bangladesh

**DOI:** 10.1186/s40733-022-00083-7

**Published:** 2022-02-09

**Authors:** Md. Abdur Rafi, Chowdhury Ibtida Tahmin, Symom Tashrik, Atia Sharmin Bonna, Ferdousy Jannat, Sabrina Jahan Mily, Abhigan Babu Shrestha, Senjuti Seemanta, Afsana Rashid, Mosarrat Mahjabeen, Nurunnahar Nura, Tasnim Shahriar, Ashrafur Rahaman Mahadi, Kawser Ahmed, Mohammad Jahid Hasan, Md. Azizul Haque, Md. Golam Hossain

**Affiliations:** 1 Rajshahi Medical College, Rajshahi, Bangladesh; 2 Pi Research Consultancy Center, Dhaka, Bangladesh; 3 Chittagong Medical College, Chittagong, Bangladesh; 4 North South University, Dhaka, Bangladesh; 5 Save the Children Bangladesh, Dhaka, Bangladesh; 6 Banshkhali Upazila health complex, Chittagong, Bangladesh; 7 M Abdur Rahim Medical College, Dianjpur, Bangladesh; 8 Shaheed Suhrawardy Medical College, Dhaka, Bangladesh; 9 Sir salimullah medical college, Dhaka, Bangladesh; 10 Khulna Medical College, Khulna, Bangladesh; 11 Central Medical College, Cumilla, Bangladesh; 12 Dhaka Medical College, Dhaka, Bangladesh; 13 University of Rajshahi, Rajshahi, Bangladesh

**Keywords:** Asthma, Inhaler, Adherence, Obstructive lung disease, Anti-asthmatic agents, Medication nonadherence, Drug compliance

## Abstract

**Background:**

Adherence to inhaler medication is an important contributor to optimum asthma control along with adequate pharmacotherapy. The objective of the present study was to assess self-reported adherence levels and to identify the potential factors associated with non-adherence to the inhalers among asthma patients.

**Methods:**

This facility-based cross-sectional study was conducted in the medicine outpatient department of Rajshahi Medical College Hospital from November 2020 to January 2021. A total of 357 clinically confirmed adult asthma patients were interviewed. Inhaler adherence was measured using the 10-item Test of Adherence scale (TAI).. Both descriptive and inferential statistics were used to express the socio-demographic of the patients and predictors of poor adherence to inhaler.

**Results:**

A substantial number of participants were non-adherent (86%) to inhaler medication. Patients non-adherent to inhaler medication are often younger (23.15, 95% CI 3.67–146.08), lived in the rural area (23.28, 95% CI 2.43–222.66), less year of schooling (5.69, 95% CI 1.27–25.44), and belonged to the middle income (aOR 9.74, 95% CI 2.11–44.9) than those adherent with the inhaler. The presence of comorbidities (12.91, 95% CI 1.41–117.61), prolonged duration of inhaler intake (5.69, 95% CI 1.22–26.49), consulting non-qualified practitioners (13.09, 95% CI 3.10–55.26) were the significant contributor of non-adherence.

**Conclusion:**

Despite ongoing motivation and treatment, non-adherence to inhalation anti-asthmatic is high and several factors have been found to contribute. Regular monitoring and a guided patient-centered self-management approach might be helpful to address them in long run.

## Background

Asthma is a heterogeneous disease, usually characterized by chronic airway inflammation, bronchial reversible obstruction, and hyperresponsiveness to direct or indirect stimuli [1]. Every year almost 495 million deaths occur worldwide from this chronic respiratory disease [2]. The prevalence is increasing by 50 % every decade, especially in the low and middle-income countries of the South-East Asian region [3, 4]. In Bangladesh, a lower-middle-income country of this region, more than eight million people are suffering from asthma that constituents almost 5.2% of the total population [5].

Successful asthma management depends on several drugs and patient-related factors like age, smoking, environmental and occupational factors, asthma-related comorbidities, choice of drug and device, patients’ adherence to the prescribed medications, and their inhaler handling techniques [6]. Inhalation therapy remains the mainstay of asthma management mostly due to its rapid onset of action, high therapeutic efficacy, and lower systemic adverse effects [6–8]. Inhaled corticosteroids along with short or long-acting beta-2 agonists and/or anticholinergic agents are most commonly prescribed as the first-line treatment for controlling asthma [8]. However, despite adequate pharmacotherapy, the control of asthma often has shown suboptimal as only prescribing appropriate medication is not adequate for achieving optimum asthma control. The Global Strategy for Asthma Management and Prevention adopted by the Global Initiative for Asthma (GINA) recommended a patient-caregiver partnership and guided self-management, along with adequate drug therapy for achieving long-term control and decreasing the frequency of exacerbation of asthma [7]. In this patient-centered approach, increasing adherence to the prescribed inhalation therapy is highly emphasized as it was evidenced that almost half of the patients with chronic diseases fail to take their long-term medications as directed at least part of the time [7, 9]. Non-adherence to the prescribed medications is an important contributor to uncontrolled asthma as well as increased healthcare utilization and increased cost [7, 10–12]. The rate of adherence varies across the country and also exists in between the age and sex groups [13]. Almost 43% of asthma patients worldwide are non-adherent to their inhalation therapy [14]. However, some studies suggest that the rate may be as high as 87% among patients with severe asthma [15, 16]. A number of personal and socio-economic factors including patients’ perception about the disease and medications, fear of side effects, patient-provider communication quality, family and social support as well as cost and availability of the drugs may influence the adherence of patients to their prescribed treatment [11, 12, 14].

Addressing non-adherence to the inhalation therapy should be a priority in the clinical assessment of asthma patients, especially those who have difficult-to-control asthma, and addressing non-adherence is likely to have greater benefits in this group than any novel treatment [17]. Despite this fact, there is hardly any evidence on adherence to asthma medication and its influencing factors among patients of Bangladesh. Moreover, using the non-validated or generalized tool for adherence assessment may invariably underestimate the incidence of non-adherence rates to the inhalers among asthma patients [9]. Hence, the present study aimed to assess self-reported adherence level and to identify the potential factors associated with non-adherence to the inhalers among asthma patients.

## Methods

### Study design and participants

This facility-based cross-sectional study was conducted in the medicine outpatient department of Rajshahi Medical College Hospital, a tertiary care referral hospital from November 2020 to January 2021. All the adult patients (aged ≥18 years) visiting the department with the diagnosis of asthma were the study population. The sample size was calculated from the following formula: $n=\frac{z^2p\left(1-p\right)}{d^2}$, where, z = 1.96 for 95% confidence level, p = assumed prevalence of poor adherence to inhaler therapy, d = allowable error of assumed prevalence. Due to lack of existing evidence, we assumed the prevalence of adherence to inhaler therapy as 50% among the asthma patients of Bangladesh, and the calculated sample size was 384. Assuming a 5% non-response rate we approached a total of 400 patients. Patients aged ≥18 years, had diagnosed asthma, and were using at least one metered-dose inhaler (MDI) with or without a spacer and/or dry powder inhaler (DPI) for at least one year were included in the study. Patients having asthma-COPD overlap syndrome, other obstructive lung diseases, chronic debilitating conditions (e.g. carcinoma), women with pregnancy, and using inhalers for less than one year were excluded. The consecutive eligible patients during the study period were recruited until the targeted number of patients was reached. After excluding the incomplete data 357 patients were included in the final analysis.

### Data collection

Face-to-face interview by five trained physicians after the consultation using a structured questionnaire and checklists was conducted to collect data from the patients and their medical records respectively. The questionnaire had four parts: (i) socio-demographic characteristics of the patients, (ii) information of inhalers they used and measurement of inhaler adherence, (iii) a demonstration session of their inhaler using technique to identify any critical error, and (iv) asthma control status using the Asthma Control Test (ACT) The questionnaire was prepared in English and translated to Bangla. Back translated version was compared with the original version to confirmed the equivalence across the language. A consortium was made to check the consistency of the translation and was pretested among 20 asthma patients before using it.

The 10-item Test of Adherence scale (TAI) based on a five-point Likert scale, which was developed and validated by Plaza et al. [18] and widely used in different countries [19, 20] were used to assess the inhaler adherence of asthma patients. However, the scale was not previously used among Bangladeshi patients, hence it was not previously validated. Patients are considered as good, intermediate, and poor adherents if they score 50, 46–49, and ≤ 45 respectively [18, 19]. In our study, poor adherence (TAI score ≤ 45) was considered non-adherent.

Patients were requested to demonstrate their inhaler using technique and were scored in a checklist adapted from a previous study based on the recommendation of the American Thoracic Society according to the steps completed by the patients to identify any critical error [2].

### Outcome and independent variables

Non-adherence to inhalers among asthma patients (TAI score ≤ 45) was the outcome variable of the present study. Independent variables were sociodemographic characteristics of the patients (age, sex, residence, educational status, family income, etc.), disease profile (smoking history, comorbidity, health-seeking behavior), and inhaler related information (type, number, and duration of inhaler usage, perceived difficulty and critical error of inhaler using technique, and self-reported efficacy of inhaler).

### Statistical analyses

All the statistical analyses were made by using STATA version 16.0. Both univariable and multivariable logistic regression models adjusted for socio-demographic and inhaler-related factors were used to determine the predictors of poor adherence to inhalers of the asthma patients. The variance inflation factor (VIF) was used to detect any evidence of multicollinearity problem among independent variables. Statistical significance level was set at *p*-value < 0.05 for a 95% confidence interval (CI).

### Ethical consideration

Ethical approval was obtained from the ethical review committee of Rajshahi Medical College to conduct the study; Memo no: RMC-IRB-2020/178. Informed written consent was also obtained from each respondent after explaining the purpose of the study.

## Results

### Characteristics of the participants

A total of 357 asthma patients were included in the study. Their mean (SD) age was 34.5 (10.2) years. Almost two-thirds of the participants were female (65%) and hailing from rural areas (62%). Almost half of them attended up to the secondary level of education and were from low-income families. MDI was the most commonly used inhaler device by the patients (75% without spacer and 13% with spacer) followed by DPI (12%). In accounts of inhaler using duration, almost 20% were using an inhaler for less than one year, 47% for 2 to 5 years, and 33% for more than 5 years. Almost half of them preferred non-qualified practitioners for their regular respiratory problems (Table 1).

**Table 1:** Sociodemographic characteristics and inhaler adherence (*n* = 357)

Characteristics	Number	Percent
Age (years) (mean = 34.54, SD = 10.18)		
18–30	148	41.46
31–40	124	34.73
> 40	85	23.81
Sex		
Male	232	64.99
Female	125	35.01
Residence		
Rural	135	37.82
Urban	222	62.18
Schooling year		
≤5	68	19.05
6–10	119	33.33
> 10	170	47.62
Family income		
Low (<BDT 15000)	196	54.90
Middle (BDT 15000 to 30,000)	138	38.66
High (>BDT 30000)	23	6.44
Smoking		
Yes	55	15.41
No	302	84.59
Comorbidity		
DM	28	7.84
HTN	41	11.48
CAD	19	5.32
Others	3	0.84
Number of inhaler		
Single	284	79.55
Multiple	73	20.45
Device		
MDI only	270	75.63
MDI + Spacer	46	12.89
DPI	41	11.48
Duration of inhaler usage (years)		
≤5	241	67.51
> 5	116	32.49
Primary care seeking behavior		
Non-qualified practitioner	166	46.50
Qualified physician	191	53.50
Demonstration of inhaler using technique		
No	127	35.57
Yes	230	64.43
Difficulty of using inhaler		
Very difficult	28	7.84
Somewhat difficult	178	49.86
Easy	151	42.30
Self-reported efficacy of inhaler		
Not effective	21	5.88
Somewhat effective	158	44.26
Effective	178	49.86
Hospital admission due to respiratory problem during last 12 months		
Yes	79	22.13
No	278	77.87
Critical error		
Yes	279	78.15
No	78	21.85
Inhaler adherence		
Overall TAI score	Mean = 36.45	SD = 7.93
Good adherence	27	7.6
Moderate adherence	21	5.9
Poor adherence	309	86.5

### Adherence to inhaler

The Cronbach’s Alpha of the TAI scale was 0.87. The mean (SD) TAI score of the asthma patients was 36.5 (7.9). The majority of the patients (86%) reported poor adherence to their inhalation therapy (TAI score ≤ 45). Almost 8% of them reported good adherence (TAI score 50) and 6% showed moderate adherence (TAI score 46–49) (Table 1). Responses to each question on the TAI scale by the asthma patients are demonstrated in Table 2.

**Table 2:** Responses to the TAI questions by the asthma patients (n = 357)

TAI questions	Responses				
	All the time, n (%)	More than half of the time, n (%)	About half of the time, n (%)	Less than half of the time, n (%)	None of the time, n (%)
How many times did you forget to take your regular inhalers in the last 7 days?	99 (27.73)	99 (27.73)	115 (32.21)	40 (11.20)	4 (1.12)
You forget to take your inhalers	86 (24.09)	87 (24.37)	75 (21.01)	101 (28.29)	8 (2.24)
	Always n (%)	Almost always n (%)	Sometimes n (%)	Almost never n (%)	Never n (%)
When you are feeling well, you stop taking your inhalers	67 (18.77)	59 (16.53)	80 (22.41)	102 (28.57)	49 (13.73)
At the weekend or when you go on holiday, you stop taking your inhalers	93 (26.05)	70 (19.61)	69 (19.33)	96 (26.89)	29 (8.12)
When you are anxious or sad, you stop taking your inhalers	134 (37.54)	108 (30.25)	60 (16.81)	35 (9.80)	20 (5.60)
You don’t take your inhalers out of fear of potential side effects	177 (49.58)	116 (32.49)	37 (10.36)	23 (6.44)	4 (1.12)
You stop taking your inhalers because you believe that they are of little help in treating your disease	196 (54.90)	82 (22.97)	47 (13.17)	30 (8.40)	2 (0.56)
You take fewer inhalations than prescribed by your doctor	85 (23.81)	53 (14.85)	100 (28.01)	99 (27.73)	20 (5.60)
You stop taking your inhalers because you believe that they interfere with your day-to-day or work life	160 (44.82)	81 (22.69)	65 (18.21)	47 (13.17)	4 (1.12)
You stop taking your inhalers because you have trouble paying for them	129 (36.13)	56 (15.69)	88 (24.65)	63 (17.65)	21 (5.88)

Multivariable logistic regression models demonstrated that younger people were more chance to be non-adherent to their inhaler therapy (aOR 23.15, 95% CI 3.67–146.08 for patients aged from 18 to 30 and aOR 8.72, 95% CI 1.62–46.85 for patients aged from 31 to 40). Besides, rural residence, less schooling year, and middle income were identified as significant predictors of non-adherence to inhaler (aOR 23.28, 95% CI 2.43–222.66 for rural residence, aOR 5.69, 95% CI 1.27–25.44 for schooling year ≤5 years and aOR 9.74, 95% CI 2.11–44.93 for middle income). Patients having comorbidities and using the inhaler for a longer period were more likely to be non-adherent to their therapy (aOR 12.91, 95% CI 1.41–117.61 for having comorbidities and aOR 5.69, 95% CI 1.22–26.49 for using inhaler > 5 years). Finally, patients visiting non-qualified practitioners (such as quacks, drug sellers, etc.) for their regular physical problems and reported inhaler using technique as difficult had a higher chance of non-adherence ((aOR 13.09, 95% CI 3.10–55.26 and aOR 10.56, 95% CI 2.95–37.73 respectively) (Table 3).

**Table 3:** Factors associated with non-adherence to inhalers among asthma patients (univariable and multivariable logistic regression models)

Variables	Non-adherence, n (%)	aOR (95% CI)
Age (years)		
18–30	140 (94.59)	23.15 (3.67–146.08)*
31–40	103 (83.06)	8.72 (1.62–46.85)*
> 40	66 (77.65)	1
Sex		
Male	206 (88.79)	1.06 (0.28–3.96)
Female	103 (82.40)	1
Residence		
Rural	131 (97.04)	23.28 (2.43–222.66)*
Urban	178 (80.18)	1
Schooling year		
≤5	62 (91.18)	5.69 (1.27–25.44)*
6–10	101 (84.87)	2.76 (0.69–11.08)
> 10	146 (85.88)	1
Family income		
Low (<BDT 15000)	155 (79.08)	1
Middle (BDT 15000 to 30,000)	134 (97.10)	9.74 (2.11–44.93)*
High (>BDT 30000)	20 (86.96)	3.57 (0.17–74.68)
Smoking		
Yes	43 (78.18)	0.51 (0.10–2.61)
No	266 (88.08)	1
Comorbidity		
Yes	42 (84.00)	12.91 (1.41–117.61)*
No	267 (86.97)	1
Number of inhaler		
Single	251 (88.38)	0.92 (0.26–3.24)
Multiple	58 (79.45)	1
Device		
MDI only	233 (86.30)	0.56 (0.14–2.22)
MDI + Spacer	43 (93.48)	2.81 (0.11–68.68)
DPI	33 (80.49)	1
Duration of inhaler usage (years)		
≤5	197 (81.74)	1
> 5	112 (96.55)	5.69 (1.22–26.49)*
Primary care seeking behavior		
Non-qualified practitioner	160 (96.39)	13.09 (3.10–55.26)*
Qualified physician	149 (78.01)	1
Demonstration of inhaler using technique		
No	117 (92.13)	1.32 (0.32–5.39)
Yes	192 (83.48)	1
Difficulty of using inhaler		
Very difficult	26 (92.86)	5.56 (0.73–42.27)
Somewhat difficult	169 (94.94)	10.56 (2.95–37.73)*
Easy	114 (75.50)	1
Self-reported efficacy of inhaler		
Effective	154 (86.52)	2.64 (0.36–19.51)
Somewhat effective	138 (87.34)	2.79 (0.35–22.36)
Not effective	17 (80.95)	1
Critical error		
Yes	256 (91.76)	1.49 (0.45–5.00)
No	53 (67.95)	1

*p-value < 0.05

## Discussion

Adherence to the inhaler and the correct using technique of the device is crucial for asthma control. Our study provides a birds’ eye view on the non-adherence to inhaler medication among the adult asthma patients of Bangladesh which exceeds 86%. Existing evidence on this issue is scarce from this country to compare with. However, some recent studies from neighboring India reported the rate of poor adherence to inhalation therapy as 71% among adults and 55% among pediatric asthma patients [21, 22]. Another study from some developing countries of Africa, like Ethiopia and Egypt, reported that almost half of the asthma patients were non-adherent to their medication [9, 23]. Though these adherence rates were also suboptimal, the situation was quite better compared to ours. However, we used the self-reported ‘Test of Adherence to Inhaler’ scale, which was a subjective assessment and might overestimate the non-adherence. The TAI test yielded high rates of poor adherence even in developed countries. For example, the ASCONA study conducted among the asthma patients of Europe reported that almost 60% of patients were poor adherent to their prescribed therapy [24]. A recent study reported an almost 58% poor inhaler adherence rate using the ‘TAI’ scale, while the rate was 29% using the pharmacy refill records, which was a more objective scale [25]. However, another study from Denmark suggested that self-reported measurements overestimate the adherence rate and might not be used as a reliable indicator [26].

A number of personal and socioeconomic factors are reported to influence inhaler adherence among asthma patients. In our study, younger people were more likely to be non-adherent irrespective of their gender. A similar phenomenon was observed in a recent meta-analysis which reported that female and younger patients are more like to be non-adherent to their inhaler therapy [14]. In our study, the rural patients belonged to middle-income families and those who were using their inhalers for a longer period had a comparatively lower adherence rate than the urban patients. In contrast to our findings, a large-scale multi-country study from European asthma patients reported no such association of these factors with inhaler adherence [13]. Though some studies suggested that patients using DPI devices had better adherence to their inhalers [27, 28], our finding did not support that. However, a very small number of patients in our study were using DPI to conclude. Besides, patients who visited non-qualified practitioners for their regular respiratory problems had more chances to be non-adherent to their therapies. A similar finding was reported by a study among inhalers using COPD patients that reported that patients who received primary care from feedback from non-qualified care providers were less sustained in medication adherence [29]. Our study suggests that asthma patients with comorbidities have a higher chance to be non-adherent. These patients showed less adherence in some previous studies too [30, 31]. Having comorbidities like diabetes, hypertension, coronary artery diseases, etc. often increase the pill burden and cost of treatment which influences the patients to be ignorant to their prescription, especially in the resource-poor socioeconomic setting. Besides these, patients’ belief and perception about the disease and its severity, self-care ability, family and social support, communication quality with healthcare providers as well as perceived efficacy of the therapy was reported as influencing factor for inhaler adherence in several studies [11, 12, 14, 32–35]. These factors were not explored extensively in our study. However, patients who reported inhaler using technique as difficult had a higher chance of non-adherence. Further qualitative studies addressing the patients’ behavioral factors and perceived barriers for inhaler adherence is necessary for better understanding. A multidisciplinary approach to support the patient both mentally and physically shared decision making between provider and patients based on possible risk and benefits could better the inhaler adherence.

The ASCONA study conducted among a large European asthma cohort reported that patients having good adherence to their inhaler therapies had better asthma control irrespective of age, sex, comorbidity and treatment modality [24]. Another large scale cohort study reported that asthma patients maintaining high adherence to their inhalers over time, had a better control of asthma [36]. Moreover, a recent review of published articles on this topic reported that good adherence to inhalers decreased the number and frequency of severe asthma exacerbations in high-quality studies [37]. Based on this evidence, it may be concluded that adherence to inhalers is a major contributor to asthma control.

### Limitations

Our study had several limitations. Firstly, it was conducted among the patients with asthma who visited the hospital outdoor for their exacerbation or other issues and so, the findings could not be inferential for the overall patient population from the community. Moreover, we used a self-reported adherence measuring tool that could potentially underestimate the non-adherence rate to inhalers as social desirability bias could not be rolled out. Some of our variables showed bizarre odds ratios in the logistic regression model which needs cautious interpretetion. Heterogeneity in the patient sample might result in these findings. More cautious inclusion criteria should be adopted in future studies. Finally, a detailed exploration of the perceived barriers of the patients was not explored extensively.

## Conclusions

Despite being an important contributing factor to asthma control, the adherence rate to the inhalers was poor among our patients. Regular investigation for patients’ adherence to the prescribed inhalers is necessary for patients with uncontrolled asthma. Adequate patient education and counseling about the nature of disease and the importance of regular use of inhalers as well as encouraging patients to seek treatment from qualified physicians is suggested to improve inhaler adherence.

## Data Availability

The data are available from the corresponding author on reasonable request.
